# Fluid intake at work in foresters working in different thermal conditions

**DOI:** 10.1038/s41598-023-41652-x

**Published:** 2023-09-23

**Authors:** J. Orysiak, M. Młynarczyk, P. Tomaszewski

**Affiliations:** 1 Department of Ergonomics, Central Institute for Labour Protection—National Research Institute, Czerniakowska St. 16, 00-701 Warsaw, Poland; 2 Department of Tourism and Recreation, Józef Piłsudski University of Physical Education, Marymoncka St. 34, 00-968 Warsaw, Poland

**Keywords:** Nutrition, Occupational health

## Abstract

The primary aim of this study was to assess the impact of fluid intake on hydration status indices in men at work. The secondary aim was to determine the type of fluids drunk at work in different thermal conditions. Fifty-nine male foresters were examined before and after one working day during summer, autumn, and winter. Before and after work, urine and blood samples were obtained from foresters. Immediately after a shift, participants completed a questionnaire regarding fluid intake during one working day. The amount of fluid consumed affects the hydration urine indices. Urine specific gravity and urine osmolality significantly decreased with increasing fluid intake (r = − 0.385 and r = − 0.405, respectively). Moreover, an impact of season on the type of fluids consumed by workers was observed. Tea was significantly more often chosen by workers to drink in winter (68%) than in summer (32%) (*p* = 0.026). The consumption of any non-alcoholic fluids contributes to the daily total water intake, but it is necessary to create individualized fluid replacement plans. Workers should know how much and what types of drinks to consume at work.

## Introduction

Water is essential for life: it provides the environment for all life processes and transports oxygen, nutrients and metabolites^1^. If the loss of water is not compensated by the intake of fluids, it leads to a decrease in the amount of water in the body. The clinical term for deficiency of total body water is dehydration^2^. Maintaining the proper hydration status has important implications for health^3^. Improper hydration status may have an impact on cognition, kidney stone risk and weight management, among other consequences^3^. For workers, dehydration can affect not only their health but also the productivity and safety of their work^3–7^. It was previously shown that an improper hydration status in workers is common both before and after work^5,8–11^. Therefore it is crucial to ensure proper hydration among workers and to monitor the amount of fluids they consume.

According to the recommendations of European Food Safety Authority [EFSA]^12^, women should consume 2000 ml of water, and men 2500 ml of water from drinks and meals. However, the requirement for water depends on many factors (diet, body composition, age, intensity and duration of work, environmental conditions, clothing, etc.); therefore even meeting the recommendations does not guarantee that workers consume enough fluids to maintain proper hydration^1,13,14^. Perrier et al.^15^ reported that low drinkers (fluid intake ≤ 1200 ml/day) had lower urine volume, higher urine specific gravity and urine osmolality, as well as darker urine than high drinkers (fluid intake 2000–4000 ml/day). Urine hydration indices may vary according the volume of fluid intake^15–18^. On the other hand, plasma osmolality was similar between the low and high volume drinkers^15^. This result may suggest that plasma osmolality was preserved by physiological adaptations despite low fluid intake^15^ and may be a good marker of acute or critical dehydration, but not of mild dehydration^5,18–20^ Thus, there is no sufficient single hydration index for all situations^5,18,21^.

Some studies have described the amount of fluid intake at work^9,22–25^. The studies showed large discrepancies in the amount of fluids consumed by workers. It should be noted that studies most often concerned only on the amount of fluid intake, while studies of the type of beverages (drinks) drunk at work have been conducted very generally^9,11,22,23,25,26^. The authors emphasized that studies on hydration status should also take into account the type of fluids consumed, not only the amount^27^.

Diet-related diseases are the leading cause of death and disability worldwide^28,29^. One of the components of the diet is water^29,30^. Beverages are the main source of water intake^29,31^, therefore their composition has a significant impact on health and disease development^29^. Depending on the content of sugar and other constituents, beverages may have different health effects^32^. High consumption of sugar-sweetened beverages (SSB) may contribute to an increase in daily energy intake, and may also reduce the quality of the diet and intake of essential nutrients^33,34^. In addition, the consumption of SSB is associated with an increased risk of e.g. type 2 diabetes, obesity, certain cancers or cardiovascular diseases^29,35,36^. In turn, the consumption of other beverages (e.g. coffee or water) in appropriate amounts has a health-promoting effect by reducing the risk of mortality, obesity, type 2 diabetes or cardiovascular diseases^37–40^. For this reason, some beverages are recommended for regular and others for occasional consumption^41^. Despite the important role of beverages in promoting or harming health, there is relatively little research on beverage consumption trends^29^.

The present study focuses not only on the amount of fluids consumed by workers, but also the frequency of choosing particular types of drinks. The study was carried out among people performing the same occupation, but in different thermal conditions (different seasons). The primary aim of this study was to assess the impact of fluid intake on hydration status indices in men at work. The second aim of this study was to determine the type of fluids drunk at work in different thermal conditions.

## Material and methods

### Participants

Fifty-nine healthy male foresters under the age of 45 (age 32.3 ± 6.9 years; height 179.7 ± 6.4 cm; body mass 90.1 ± 15.4 kg; body mass index (BMI) 23.7 ± 6.3 kg/m^2^) participated in the study. A forester manages, organizes and supervises works in the field of forest management. He does most of his work in the forest, covering many kilometres every day in various thermal conditions during different seasons. A forester should be in good physical condition, orientated in the field and resistant to low and high temperatures^42^. The specific nature of a forester’s work tends to promote dehydration of the body, therefore this group was selected for the study. The study was conducted in the workplace at different seasons of the year. None of the volunteers experienced gastrointestinal problems (diarrhoea, vomiting, and constipation) during the study.

This study includes some data previously presented^5,24^, but the current aims and analyses differ from the mentioned work.

The study was conducted in accordance with the Declaration of Helsinki and approved by the local ethics committee.

### Study design

Foresters were examined before and after one typical working day during three seasons of the year (summer, autumn, and winter). Before (1) and after (2) work, urine and blood samples were obtained from foresters. Also, the body mass and total body water were determined before and after work. The same hydration status indices were measured during each timepoint. Immediately after a shift, participants completed a questionnaire regarding fluid intake during 1 working day. Each participant was tested only once during one season of the year. The work shift lasted at least 6 h^5,24^.

### Hydration status indices

The following hydration status indices were used: thirst, frequency of urination, body mass (BM), total body water (TBW), urine colour (Ucol), urine specific gravity, urine osmolality, serum osmolality (Sosm), percentage change of body mass (%BM), percentage change of total body water (%TBW) and percentage change of serum osmolality (%S_osm_)^7,19,20,43^. Urine samples were collected before work (in the morning, in the fasting state, during the first morning voiding) and after work (when urine was in the bladder for approx. 2 h before sample collection). Similarly, blood samples were taken before work (fasting) and after work from the fingertip (capillary blood). Urine-specific gravity was determined at the workplace, while samples for urine and serum osmolality measurement were frozen (at − 20 °C), and these markers were determined in a diagnostic laboratory^5^. Laboratory tests were carried out by qualified personnel.

The tested hydration status indices along with a description of the method used to determine them were are follows^5^:

- Thirst—questionnaire.
- Frequency of urination at work—questionnaire.
- Body mass (BM)—Inbody 270 body composition analyser (Biospace, Cerritos, California, USA).
- Total body water (TBW)—Inbody 270 body composition analyser (Biospace, Cerritos, California, USA).
- Urine colour (Ucol)—8-point scale^44^.
- Urine specific gravity—PAL-10S Atago refractometer (Tokyo, Japan).
- Urine osmolality—cryoscopic method using a Marcel OS3000 osmometer (Poznań, Poland).
- Serum osmolality (S_osm_)—cryoscopic method using a Marcel OS3000 osmometer (Poznań, Poland).
- Percentage change of body mass (%BM) calculation method according to the formula.
- Percentage change of total body water (%TBW) calculation method according to the formula.
- Percentage change of serum osmolality (%S_osm_) calculation method according to the formula.

The percentage change of specific indices (%BM, %TBW, %S_osm_) was calculated as^5,9^:

$$\text{Percentage change}= \frac{\text{marker of hydration status after work }\left(2\right)- \text{marker of hydration status before work} \left(1\right)}{\text{marker of hydration status before work }\left(1\right)} \times 100\%$$

Dehydration was defined according to the urine specific gravity ≥ 1.020 g/mL^5,7^. A detailed description of the used methods can be found in a previous study^5^.

### Questionnaire analysis

Data on the total water intake from fluids and foods were obtained from questionnaire studies. Immediately after a shift, participants completed a questionnaire regarding fluid intake during one working day^24^. The questionnaire created by the authors asked about volume and type of fluids, volume of soups, and type and amount of fruits and vegetables consumed by workers at work. Questions about fluids included: water (bottled, tap, sparkling, still), juices, milk and dairy products, sodas (carbonated drinks without sparkling water), coffee, tea and infusions, alcoholic beverages, alcohol free beer, energy drinks, sports drinks, plant-based beverages, meal replacement drinks (including protein shakes and supplements) and others. Due to the small amount of specific type of fluids consumed by workers, the category “others” included: alcoholic beverages, non-alcoholic beer, plant-based beverages, isotonic beverages, meal replacement beverages (including protein shakes, supplements) and others.

Later in the article water intake comes from fluids (water from fluids) including beverages (drinks) and drinking water. The water content from beverages and water, as well as soup, was calculated based on a specific volume of fluids consumed. The water content from fruits and vegetables was calculated using the “*Tabele składu i wartości odżywczej żywności*” (Tables of food composition and nutritional value)^45^ considering the portion size. All calculations (fluids, soup, fruits, and vegetables) were performed separately and expressed as millilitres of water per shift. Total water intake (TWI) was calculated by adding up the amount of water from fluids, the amount of water from fruits, the amount of water from soups and the amount of water from vegetables.

### Severity of work

The severity of the work was determined on the basis of the Borg Rating of Perceived Exertion (Borg RPE Scale®) scale^46,47^. This scale measures the overall intensity of physical activity based on the physical sensations experienced by a person during physical activity^48,49^. In the present publication, the scale was used to measure the severity of the work performed by foresters (from a rating of 6—“no exertion at all”—to 20—“maximal exertion”).

### Environmental conditions

The values of air temperature prevailing on the days of the study, measured at the same time (mid-shift) outdoors, in the summer, autumn and winter period in Poland were 20.5 ± 0.0 °C; 16.8 ± 0.0 °C and 4.3 ± 0.0 °C, respectively^5,24^.

### Statistical analysis

Basic statistical measures were used to describe the results: arithmetic means, standard deviations (SD), and percentages. The assumption of normality of the distributions of analysed variables was tested using the Shapiro–Wilk test, whereas homogeneity of variance was verified using the Levene test. Due to severe violation of the above assumptions, the non-parametric procedures of the Freidman test and Wilcoxon test, as well as the Kruskal–Wallis test, were used in the analysis. The chi-square test of maximum likelihood and McNemar's test were used to compare frequency of consumption and numbers of workers who changed their hydration status, respectively. The relationships between variables were tested using Spearman’s rank correlation. Analyses were performed using STATISTICA 13 (TIBCO Software, Palo Alto, CA, USA). The level of *p* < 0.05 was set to evaluate the significance of the effects.

### Ethical approval

The study was conducted in accordance with the Declaration of Helsinki and approved by the Local Ethics Committee of the Institute Of space Sport-National Research Institute (protocol code KEBN-21-63-JO).

### Consent to participate

Informed consent was obtained from all individual participants included in the study.

## Results

### Correlations between water intake and hydration status indices

In this section, the results of hydration status indices presented previously^5^ were used. There was a significant correlation between water intake from fluids, total water intake, and some hydration status indices. The amount of water consumed from fluids at work positively correlated with the percentage change in body mass from after work to before work (%BM 2 to 1; r = 0.317; *p* = 0.015) and the percentage change in total body water volume from after work to before work (%TBW 2 to 1; r = 0.267; *p* = 0.041). On the other hand, urine specific gravity and urine osmolality significantly decreased with increasing fluid intake (r = − 0.385; *p* = 0.003 and r = -0.405; *p* = 0.001, respectively). For total water intake, the higher the total water intake, the greater the percentage change in body mass after work to before work (%BM 2 to 1; r = 0.359; *p* = 0.005) as well as the percentage change in total body water volume from after work to before work (%TBW 2 to 1; r = 0.342; *p* = 0.008). Conversely, when total water intake decreases, urine specific gravity and urine osmolality increase (r = − 0.372; *p* = 0.004 and r = − 0.358; *p* = 0.005, respectively). However, water consumed from fluids and total water intake were not significantly correlated with participants’ BMI, serum osmolality, changes in serum osmolality, urine colour or perceived thirst at work. The amount of water consumed from vegetables was found to positively correlate with the percentage change in total body water volume from after work to before work (%TBW 2 to 1; r = 0.363; *p* = 0.005). Moreover, no significant correlations were found either for water consumed from fruits or from soups and hydration status indices.

A significant (*p* = 0.033) relationship was observed between the frequency of urination at work (2.4 ± 1.2; min = 1; max = 5) and the volume of fluid intake at work (r = 0.279). The more fluid is consumed, the more frequent is urination at work.

The mean perceived severity of the work was 10.8 ± 2.0 (min = 6; max = 15). No significant relationship was observed between the severity of the work performed and the amount of water consumed from fluids (r = 0.003; *p* = 0.983) or the total water intake at work (r = − 0.112; *p* = 0.428).

### Total water intake

The mean amount of water from fluids at work (regardless of the season) was 931 ± 496 ml/work shift (min. 250 ml/work shift; max. 2500 ml/work shift). The mean total water intake at work was 1126 ± 574 ml/work shift (min. 250 ml/work shift; max. 2686 ml/work shift). The intake of water from fluids, as well as the total water intake, varied between seasons (Table 1). In autumn, the intake of water from fluids at work was significantly lower than in summer (*p* = 0.034). Similarly, in autumn the total water intake was significantly lower than in summer (*p* = 0.026). There were no differences in the intake of water from vegetables, fruits and soups between seasons (Table 1). In summer, approximately 83% of water intake comes from fluids (including drinking water and beverages), 5% from fruits and 12% from vegetables. In autumn, approximately of 79% of water intake comes from fluids (including beverages and drinking water), 3% from fruits, 12% from soup and 7% from vegetables. In winter, approximately of 86% of water intake comes from fluids (including drinking water and beverages), 2% from fruits and 12% from vegetables (Table 1).

**Table 1:** Total water intake of foresters (n = 59) at work, along with the percentage of water source to total water intake (mean ± SD).

Water source	Season of the year at work on the test day		
	Summer	Autumn	Winter
Water [ml]^a^	603 ± 628 51%	293 ± 293 39%	237 ± 230 27%
Juices [ml]^a^	126 ± 220 11%	26 ± 109 3%	103 ± 183 12%
Milk [ml]^a^	11 ± 27 1%	17 ± 76 2%	34 ± 105 4%
Carbonated drinks [ml]^a^	108 ± 239 9%	69 ± 138 9%	76 ± 235 9%
Coffee [ml]^a^	145 ± 187 12%	217 ± 231 29%	187 ± 192 21%
Tea [ml]^a^	158 ± 364 13%	112 ± 160 15%	208 ± 161 24%
Energy drinks [ml]^a^	39 ± 95 3%	12 ± 55 2%	0 ± 0 0%
Others [ml]^^^^a^	0 ± 0 0%	7 ± 33 1%	26 ± 81 3%
Water from fluids (drinking water and beverages) [ml]^b^	1189 ± 592* 83%	752 ± 403 79%	871 ± 388 86%
Water from fruits [ml]^b^	76 ± 115 5%	25 ± 53 3%	20 ± 43 2%
Water from soup [ml]^b^	0 ± 0 0%	110 ± 189 12%	0 ± 0 0%
Water from vegetables [ml]^b^	169 ± 226 12%	66 ± 90 7%	118 ± 117 12%
Total water intake [ml]	1435 ± 634*	952 ± 527	1009 ± 449

^Other (alcoholic beverages, non-alcoholic beer, plant-based beverages, isotonic beverages, meal replacement beverages and others). *Significantly (*p* < 0.05) different from autumn.

^a^Water intake from particular beverages was calculated as a percentage of a specific type of beverage in the total amount of water derived from beverages and drinking water (water from fluids).

^b^Water intake from various source was calculated as a percentage of water from fluids/fruits/soup/vegetables in the total water intake.

### Percentage of water from fruit and vegetables

Regardless of the season, workers obtained an average of 13% (± 14%) of water from fruits and vegetables. Regarding season, the percentage of water consumed with fruits and vegetables was in summer 17 ± 20% (min 0%; max 63%), in autumn 9 ± 10% (min 0%, max 29%), and in winter 13 ± 11% (min 0%, max 34%).

### Type of fluids

The largest percentage of workers, regardless of the season, consumed water, followed by coffee and tea. In summer, the largest percentage of workers chose water (79%) and coffee (47%), in autumn the most workers drank water (67%) and coffee (57%), and in winter water (68%) and tea (68%) (Table 2). Tea was chosen by workers significantly more often in winter than in summer (*p* = 0.026) and nearly significantly as compared to autumn (*p* = 0.058).

**Table 2:** Percentage of foresters (n = 59) who chose certain types of fluids.

Season of the year	Type of fluids							
	Water (%)	Juices (%)	Milk (%)	Carbonated drinks (%)	Coffee (%)	Tea (%)	Energy drinks (%)	Other^ (%)
General	71	22	10	24	54	46	7	5
Summer	79	32	16	32	47	32	16	0
Autumn	67	10	5	24	57	38	5	5
Winter	68	26	11	16	58	68*	0	11

^Other (alcoholic beverages, non-alcoholic beer, plant-based beverages, isotonic beverages, meal replacement beverages and others). *Significantly (*p* < 0.05) different from summer.

### Change in hydration status before work versus after work

In this section, the urine specific gravity results presented in the previous paper^5^ were used. Table 3 shows the change in hydration status of volunteers from ‘before work’ to ‘after work’, assessed on the basis of urine specific gravity. The mean urine specific gravity before work was significantly (*p* < 0.001) higher than after work (1.022 ± 0.006 vs. 1.018 ± 0.006 g/m). Thirty-eight (64%) of the 59 workers were dehydrated before work and 30 (51%) after work. Volunteers who were dehydrated at the beginning and end of work, and those who were properly hydrated (euhydrated) before work but dehydrated at the end, consumed less fluid than those who were normally hydrated after work, but these differences did not reach statistical significance. Additionally, no significant differences in numbers of workers whose hydration status changed was observed (McNemar's test = 2.04; *p* = 0.15).

**Table 3:** Change in hydration status in foresters (n = 59) from beginning to end of the work according to urine specific gravity.

Hydration status	Fluid intake at work [ml]	Number (percentage) of workers with a change in their hydration status
Euhydrated to euhydrated	1150 ± 603	13 (22)
Euhydrated to dehydrated	600 ± 266	8 (14)
Dehydrated to dehydrated	859 ± 468	22 (37)
Dehydrated to euhydrated	1019 ± 453	16 (27)

## Discussion

The results of this study suggest that urine hydration indices are influenced by the amount of fluids consumed by workers. Urine specific gravity, as well as urine osmolality, correlated with water intake from fluids and total water intake. When water intake from fluids as well as total water intake decrease, urine specific gravity and urine osmolality increase; i.e. lower water intake may promote dehydration. Moreover, workers most often consumed water, coffee and tea, regardless of the season. In this study, the impact of the season on the type of fluids consumed was observed. Tea was significantly more often chosen by workers to drink in winter than in summer. Fruit and vegetables can also provide a source of water for workers. In addition, there was considerable variation in the amount of fluids consumed among workers. Such results may indicate a lack of knowledge among workers about the personalized fluid intake depending on their needs. This underlines the need to draw workers’ attention to the amount and type of fluids they consume and their impact on health. At work, workers should consume water, unsweetened coffee or tea, which have health-promoting effects. On the other hand, drinking sugary drinks can negatively affect workers’ health^41^. Similarly, not drinking enough fluids can not only increase the risk of heat stress, but also impair work capacity^50^.

### Correlations between water intake and hydration status indices

Urine hydration status indices may vary depending on the amount of fluids consumed^15,18^. Lower urine volume, higher urine specific gravity and osmolality and darker urine colour were observed in drinkers of lower amounts of fluids (≤ 1000–2000 ml/day) than drinkers of higher amounts of fluids (2000–4000 ml/day)^15^. Similarly, a relationship between water intake and hydration status (defined by urine specific gravity) was observed in production workers in a fish processing company^51^. Workers who drank less than 1475 ml had 3.750 times higher risk of being dehydrated than workers who consumed more than 1475 ml^51^. Perrier et al.^18^ also observed that total fluid intake volume (from food and fluid) correlated with 24-h urine osmolality, colour, specific gravity and volume, as well as solute concentration. Of note, a strong correlation between urine hydration markers and fluid intake was found in 24-h urine, but not in first morning urine^18^. It was shown, that urine volume increase and urine osmolality and specific gravity decrease with increasing fluid intake volume^18^. Similarly, in our study, urine specific gravity and urine osmolality significantly decreased with an increasing volume of fluid intake, as well as total water intake. These results are consistent with previous suggestions that multiple factors (e.g. fluid intake, diet, exercise, ambient temperature, and the time from the previous voiding) influence urine hydration status indices^43,52^. Therefore, it should be noted that post-work hydration status is most likely to reflect acute changes in hydration status, not the current hydration status. For a more accurate assessment of hydration status, it is better to measure hydration status indices in the first morning urine^5,52^. Moreover, it was found that more than 50% of the variance of fluid intake volume may be explained by urine volume^18^. In our study, we did not accurately measure the urine volume, but we observed an increase in the frequency of urination at work as the fluid intake increased. It was also found that fluid intake, as well as total water intake, positively correlated with the percentage change in body mass (%BM 2 to 1) and the percentage change in total body water volume (%TBW 2 to 1). These results confirm our previous observations in which an increase in body mass and total body water was observed after work^5^. Such a result may suggest that foresters did not adjust the amount of fluid intake to current needs (they drank too much fluid). It is also not in line with the recommendations according to which the amount of fluids consumed should not cause a significant increase in body weight^53^. It follows that workers should create their own hydration plans^54,55^.

### Total water intake

There are not many studies on the effect of various environmental conditions on water balance. Although water balance in summer and winter was similar^56^, water intake and water loss were higher in summer due to intense sweating caused by high temperature (heat stress)^56^. It was also observed that work environmental temperature had an impact on hydration status^51^. The risk of dehydration is 9.305 times higher at work environmental temperature ≥ 28 °C WBGT (welt bulb globe temperature) than at < 28 °C WBGT^51^. Therefore the amount of water consumed from fluids may vary between seasons of the year. Our findings show that foresters consumed more water from fluids in the summer (20.5 ± 0.0 °C) than in the autumn (16.8 ± 0.0 °C). Moreover, the total water intake may vary between workers in different seasons. In this study, the total water intake at work was higher in the summer than in the autumn. Malisova et al.^56^ also observed that in the general population in Greece the total water intake differed between summer and winter (mean temperature in individual months in summer was 34.9 °C and 35.4 °C, and in winter 13.5 °C, 14.2 °C and 14.6 °C). The total water intake in summer was about 40% higher than in winter^56^. A study of loggers (forest workers) also showed that they consumed more fluids in summer (WBGT 16.3 ± 4.3 °C) than in winter (WBGT 8.8 ± 2.8 °C)^25^. Although similar relationships were observed in the total water intake between different seasons of the year, it should be noted that in each of these studies a different questionnaire was used to assess water intake. The method of determining the air temperature also differed.

In this study the mean intake of water from fluids at work (regardless of the season) was 931 ± 496 ml/work shift (min. 250 ml/work shift; max. 2500 ml/work shift). The average fluid intake at work in our study appears to be insufficient, as more than 50% of workers were dehydrated after work. Bates et al.^22^ observed that most loggers in New Zealand did not drink enough fluids in the first five hours of work (mean 1124 ± 822 ml). On the other hand, the mean fluid intake per shift of industrial workers under thermal stress was 6480 ml, which protected workers from “involuntary dehydration”^23^. In turn, UAE construction workers drank on average 5440 ml per 12-h shift, which was enough to maintain an optimal hydration status^26^. Differences in fluid consumption in in this study and in our previous report^24^ compared to others could be attributed to various factors such as environmental conditions, lack of hydration education, work severity, and shift duration.

Parker et al.^25^ suggested advising workers to drink enough to pass urine regularly (at least once during work), as well as to have pale/light urine colour rather than prescribe a volume (amount) of fluid to consume during the work. Depending on the season, in the present study, the minimum amount of fluids consumed by foresters was in summer 250 ml, in autumn 250 ml and in winter 350 ml. The maximum amount of fluids consumed by foresters was 2500 ml in summer, 1800 ml in autumn and 1800 ml in winter. The present study results are consistent with our previous pilot study, which also showed differences between workers in the amount of fluids consumed at work between summer and winter^24^. In a previous study, in the summer, the amount of fluids drank by foresters ranged from 600 to 2150 ml, and from 350 to 1800 ml in the winter^24^. Similarly, Bates et al.^22^ observed differences in the amount of fluids consumed among loggers (min 0 ml, maximum 3000 ml in the first five hours of work). Biggs et al.^9^ also reported differences in the volume of fluid intake, some forestry workers did not consume fluids during the shift at all. In our study all foresters drank fluids at work, although the amount varied. Such large variation in the intake of fluids among foresters most likely reflects the variation in individual fluid requirements. It is also worth emphasizing that reaching the recommended level of water intake does not guarantee that workers drink the amount of fluids they need^13^.

### Percentage of water from fruit and vegetables

It was found that 24% of water intake comes from solid foods in winter and 15% in summer^56^, which confirmed that water from food is a significant contribution to daily water intake (total water intake)^56^. Moreover, a significant contributor of TWI was water from food (40.9–61.3%) also in young adults in China^27^. In the present study, the amount of water from fruits and vegetables was on average 13% (± 14%). However, large discrepancies were noted. Some foresters did not consume any fruit and vegetables at work, while in others more than 60% of the total water intake came from fruit and vegetables.

Given that a significant amount of fluid intake occurs during meals^57,58^, workers should be encouraged not to skip meals^4,59^. However, not all studies confirm this. In the study of Brake and Bates^23^ beverage consumption during meal breaks was 31,000 ml (which is 14% of total fluid intake). The lower intake of water during meal breaks may be due to ad libitum access to cold water (also with cordial flavouring) on the job, as well as workers being well educated regarding the amount and frequency of drinking^23^. It is worth noting that meals (meal breaks) may not significantly affect the amount of fluid intake for properly hydrated workers, but meals may be an important source of minerals, e.g. those lost with sweat^23^. However, if participants are dehydrated and have lower total water intake, they are unlikely to compensate for their water needs from water from food^27^.

### Type of fluids

In this study, we focused on the drinking preferences of workers, a topic that holds significant implications for productivity, wellbeing, and health. Water, coffee, and tea emerged as the top beverage choices, confirming findings from previous study of Guelinckx et al.^60^.

Seasonal changes influence drinking habits. More workers consumed tea in winter than in summer, possibly due to the need for warmth in colder temperatures. This aligns with recommendations for workers to consume chilled beverages in hotter climates and warm drinks in cooler climates^13,61–63^. However, these findings should be contextualized within individual preferences and external factors such as climate and work conditions. In line with the present study, the influence of season on the types of drinks consumed by workers was also observed by Parker et al.^25^. They reported the differences in the type of beverages consumed at work between summer and winter in loggers: 54% of loggers consumed only tea of coffee, 15% drank soup or hot non-caffeinated beverages, and 5% drank only water in winter^25^. In contrast, during summer 46% of loggers drank tea or coffee, 28% consumed both tea/coffee and water, 13% drank cordial and 9% consumed only water^25^. Malisova et al.^56^ observed that 61% and 50% of water intake came from drinking water, in summer and winter, respectively. Regarding water intake from beverages, 9% and 9% from tea, as well as 33% and 25% from coffee, were noted in the Greek population in summer and winter, respectively^56^. Industrial workers working 10–12.5 h under thermal stress (mean WBGT 30.9 ± 2.0 °C) drank mainly water with relatively minor intake of coffee, tea, cola soft drinks and sports drinks^23^. In a study of agricultural workers in Oregon and Washington, it was observed that 65 versus 31% of workers consumed sodas, 69 versus 23% sports drinks, and 41 versus 8% juices, respectively^64^. Concerningly, we observed that around one-third of forestry workers consumed fruit/vegetable juices and sweetened carbonated drinks in the summer. High sugar beverage consumption can have detrimental health effects^29,35,36^, and educational programmes on proper workplace hydration are needed^22^.

Although 100% fruit juices may be seen as a healthy choice because they contain some vitamins and minerals, they are also high in natural sugars. Research into the health effects of different types of juice requires more studies, and it is currently recommended to limit drinking of juice to 1 cup per day^65,66^. In turn, sweetened beverages have a high content of added sugars that result in high daily energy intake^33^. Among the EU Member States, 20% of people in Belgium, and around 12% in Malta, Germany, Hungary, Poland and Bulgaria, reported drinking sugar-sweetened soft drinks at least once a day^67^. Moreover, on average 6.5% of U.S. adults’ total daily calories comes from sugar-sweetened beverages^68^. This means that the recommended daily intake of free sugars (added sugars plus sugars that are naturally present in honey, syrup and fruit juices, no more than 10% of total energy intake) may be exceeded by consuming sweetened carbonated beverages alone^29,36,69^.

However, advice to avoid specific drinks (e.g., those containing caffeine) could inadvertently result in lower total fluid intake, especially if those beverages are liked by workers and part of their normal diet ^21,70^.Therefore, workers should have a certain “autonomy” regarding the type of fluids consumed^71^, but knowing about the impact of different drinks on health will allow them to choose health-promoting drinks.

### Change in hydration status before work versus after work

It was observed that most UAE construction workers arrived at work euhydrated and maintained this status over 12-h shifts^26^. Underground miners with optimal hydration before work remained hydrated after work (pre-shift to post shift)^11^. Hydration status is a critical concern for workers. In our study, only 22% of forestry workers who were properly hydrated before work maintained this state afterwards. In turn, if the workers were dehydrated before a shift they were 2.6 times more likely to be poorly hydrated after the shift compared to those who arrived at work in good hydration ^11^. Nurses and doctors who were dehydrated at the end of the shift had a mean volume of fluid intake approximately 30% less than euhydrated workers at the end of the shift^10^. Moreover, 70% of workers who were euhydrated before work were dehydrated after work, and 57% of workers who were dehydrated before and after work drank less than 1475 ml^51^. In present study of foresters, workers who were dehydrated after work consumed less fluids at work than workers who were not dehydrated after work, but this was not statistically significant. It appears that inadequate fluid intake, restricted break times, and lack of awareness about hydration may contribute to dehydration at work^4,10^.

### Limitations

This study has some limitations. Our sample size was small and consisted solely of male foresters, which might limit the generalizability of the results. However, our research’s strength lies in its focus on a single-gender population performing the same occupation across different seasons, limiting confounding variables. Our findings contribute to understanding worker hydration behaviours, which can inform interventions to promote healthier drinking habits and improve worker well-being.

## Conclusion

In conclusion, our study underscores the significant influence of fluid intake on the hydration status of workers, specifically foresters. Notably, laboratory assessments of post-work hydration may not exactly mirror overall hydration status but may rather indicate acute changes in fluid intake. Importantly, the quantity of water consumed by workers does not necessarily align with their individual hydration needs.

Our findings also reveal the impact of seasonal changes on the type of beverages workers choose, underscoring the necessity for appropriate education on fluid consumption at the workplace.

With these insights in mind, workers should consider the following guidelines:

- Water is the ideal choice for hydration, due to its absence of sugars and calories.
- Chilled drinks are preferred in warmer climates, while warmer ones are more suited to colder temperatures.
- All beverages can influence hydration status, but their health impacts can vary. Drinks high in sugar and calories, for instance, can have harmful effects.
- Fluid intake should be personalized, as water requirements depend on numerous factors such as the intensity and duration of work, environmental conditions, age, body composition, and body mass.
- To ensure adequate hydration, workers should drink enough to use the toilet at least once during their shift and their urine should be light in colour.

Our study provides valuable insights to improve workplace hydration practices, ultimately contributing to worker health and productivity.

## Data Availability

All data are incorporated into the article.
